# Delayed autotransplantation as a method of single defect treatment with sinus perforation. Case report

**DOI:** 10.4317/jced.59679

**Published:** 2023-02-01

**Authors:** Igor Ashurko, Ulvi Shirnaliev, Voronov Ulvi, Leyli Orazova

**Affiliations:** 1 Surgery Dentistry, Sechenov First Moscow State Medical University of the Ministry of Health of the Russian Federation (Sechenov University), Moscow, Russia; 2 Private Dental Clinic Belgravia Dental Studio, Moscow, Russia

## Abstract

Autotransplantation of teeth as an alternative to dental implantation is characterized by short healing periods, preservation of aesthetics and propriosensitivity in the area of the transplanted tooth and the possibility of its orthodontic movement. This clinical case describes a successful delayed autotransplantation of the third maxilla molar (2.8.) with the completed root formation into the socket of a previously removed tooth 1.6. with perforation in the maxillary sinus area on the right and the presence of signs of chronic inflammation. Long-term observations after 30 months demonstrate favorable healing with restoration of dentoalveolar attachment in the area of the transplanted tooth, relief of inflammatory process in the area of the maxillary sinus with restoration of the cortical plate.

** Key words:**CBCT, tooth transplantation, dental autotansplantation, wisdom teeth.

## Introduction

Despite the fact that today dental implantation is one of the priority methods of treating the absence of teeth, the possibility of its use depends on a number of conditions. One of these conditions is the state of the bone tissue in the area of the planned intervention. Bone tissue deficiency can be caused by a plenty of reasons: traumatic tooth extraction, extensive inflammatory process, close location of the maxillary sinuses, etc.

Obviously, performing bone regeneration techniques increases the risk of possible complications (1), as well as increases the traumatic nature of the intervention, the duration and cost of rehabilitation.

An alternative method for the treatment of single defects of the dentition is considered to be autotransplantation of the tooth, which becomes a predicTable option for the restoration of chewing and aesthetic function (2,3). Up to now, quite a lot of experience in autotransplantation has been accumulated, but clinicians do not often turn to this technique, due to the existing possibility of getting ankylosis with subsequent replacement tooth tissue resorption, so it is currently used as an alternative treatment for single defects (2,4).

This article describes the method of autotransplantation of a tooth in the case when dental implantation would be associated with a longer and more complex treatment.

## Case Report

-Patient information

A 35-year-old patient came to the clinic 7 days after tooth extraction 1.6, complaining about the presence of communication of the oral cavity with the maxillary sinus, difficulty in eating. There were neither relevant medical problems or allergies. The family history was unremarkable.

-Clinical findings 

While the examining the oral cavity, the socket of the extracted tooth 1.6. filled with a fibrin clot was determined. The sutures were well-maintained, covered with a soft plaque. Oral-nasal and nasal-oral tests were positive. When probing the distal surface of the tooth 1.5. a periodontal pocket with a depth of 10 mm was determine. The mucous membrane surrounding the tooth had pale pink color, without pathological changes and signs of inflammation. There was no discharge from the socket. There were no clinical signs of acute maxillary sinusitis.

-Diagnostic assessment

**Figure 1:**
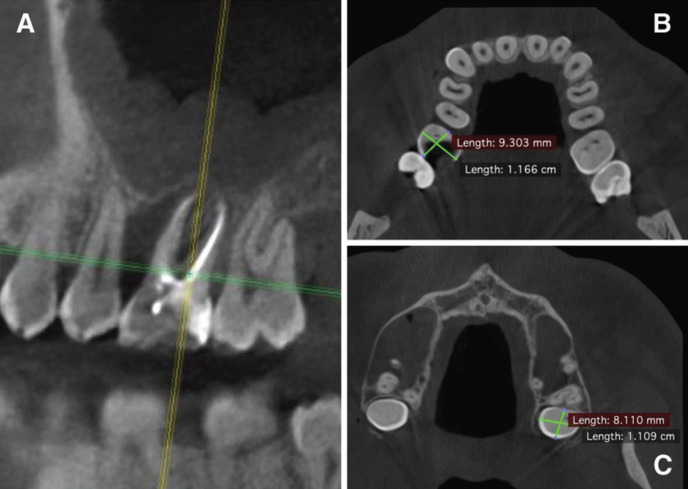
A. CBCT scans before surgery, showing vertical bone loss and inflammation in the maxillary sinus. B. Measurements of the extracted tooth. C. Measurements of the donor tooth.

The analysis of cone-beam computed tomography (CBCT) revealed a local vertical loss of bone tissue along the distal surface of the tooth 1.5, the absence of a part of the vestibular cortical plate in the area of the socket of the extracted tooth 1.6. and the bottom of the maxillary sinus on the right, as well as signs of parietal thickening of the mucous membrane in the area of the bottom of the maxillary sinus (Fig. 1A,B). Also, during the diagnosis, a retainted tooth 2.8 was revealed, dystopian in the buccal side (Fig. 1C). Based on the data of clinical examination and radiation examination, chronic maxillary sinusitis was diagnosed (J.32.0).

-Treatment planning

The patient was offered several options for restoring the integrity of the dentition: fabrication of a bridge prosthesis, dental implantation and autotransplantation of the tooth. Due to the potential complexity of rehabilitation with the use of a dental implant, as well as the presence of a wisdom tooth (2.8), which is not of functional value, the patient chose a treatment plan that includes autotransplantation of the tooth.

Surgical intervention

**Figure 2:**
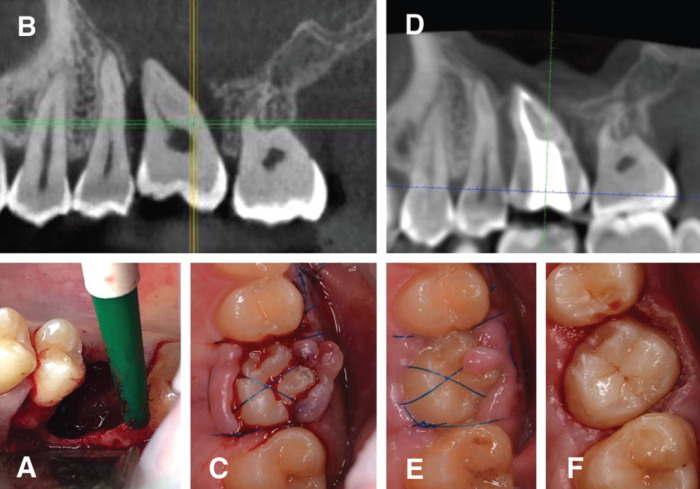
A. Revealing of a local vertical bone tissue loss. B. CBCT scans after surgery. C. Situation after suturing. D. CBCT scans after endodontic treatment. E. Condition of soft tissue, 14 days after surgery. F. Soft tissue excision made by diathermic coagulator.

10 days after extracting 1.6 tooth, patient was prescribed with amoxicillin/clavulanic acid 875mg/125mg 1hour prior to surgery. Under local anaesthesia with 4% articaine hydrochloride plus 1:100,000 epinephrine, sulcular incisions were made in the teeth area 1.5, 1.7, vertical incision in the tooth area 1.5. The muco-periosteal flap was elevated from the vestibular surface to the borders of the vestibular cortical plate bone defect (Fig. 2A). Careful curettage of the socket of the extracted tooth 1.6 was performed. Perforation of the maxillary sinus was detected along the medial buccal socket of the extracted tooth. Abundant rinsing with 0.05% chlorhexidine gluconate and sterile saline was performed to eliminate debris. Tooth 2.8 was gently extracted with dental elevator and examined and examined for root damage. Then the tooth was immediately placed into the recipient site and splinted with nodular and cruciform sutures Polypropylene 6-0 (Fig. 2B,C). The occlusal adjustment of the donor tooth was performed to remove any interferences.

After surgery, the patient was prescribed with amoxicillin/ clavulanic acid 875mg/125mg two times per day for 5days and nimesulide 100mg when necessary. Patient was instructed to rinse with 0.2% chlorhexidine gluconate two times per day for a week.

Sutures were removed after 14 days postsurgery. During the objective examination the soft tissue proliferation was visualized (Fig. 2E). Then the soft tissue excision was made with the use of diathermic coagulator in order to isolate the tooth (Fig. 2F). Endodontic treatment was performed on the 14th day postsurgery. The root canals were sealed with a paste based on calcium hydroxide, and also obturated with gutta-percha pins, two and three weeks after the operation, respectively (Fig. 2D).

After 3 months of follow-up, the transplant was functional without any discomfort, mucosa has physiological color, no sensitivity to percussion, no pathologic mobility and no symptoms of any inflammation appeared and thus a prosthetic treatment was received by ceramic restoration.

-Outcome and Follow up

**Figure 3:**
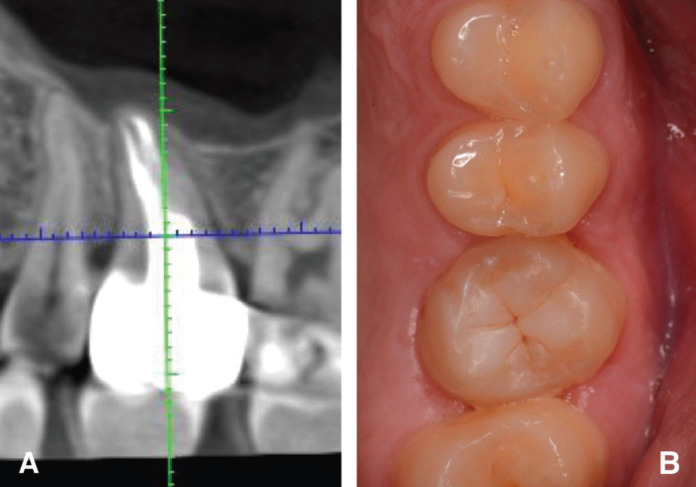
A. CBCT scans after 30 months postsurgery. B. Condition of soft tissue, 30 months after surgery.

6 months after the treatment, the patient had no complaints, the mobility of the tooth was determined within the physiological norm. The depth of probing of the dentoalveolar sulcus is within 2-3 mm. In 30 months, clinical and radiological data showed the stability of the treatment result, complete regeneration of bone tissue, and the absence of inflammatory process from the maxillary sinus (Fig. 3A,B).

## Discussion

Caries and its complications are the most common reasons for tooth extraction. Dental implantation is considered to be the most acceptable solution for the recovery of a tooth loss, but often its implementation in the distal parts of the jaw is complicated by the need of plastic bone surgery

As a rule, bone grafting is a solution to the problem of bone deficiency. Bone atrophy can be caused by prolonged absence of teeth(5), prolonged inflammatory process, traumatic tooth extraction(6), anatomical features of the patient, such as the proximity of the maxillary sinuses.

Thus, in almost half of the cases (54,6%), the maxillary sinus was located in the molar area (7) and according to Davide Rancitelli or George Psillas the implantation in this area that is located close to the maxillary sinuses is a complicated task for surgeons (8), which is followed up by different complications.

Implantation is often impossible or involves great risks at each stage of treatment: the presence of chronic inflammation, perforation of the sinus, in the process of removal, traumatization of the vestibular wall of the alveolar process. Referring to Esposito M, Felice P., Worthington HV, the probability of complications after sinus lifting increases by 4.77%(9). Often the bone tissue atrophy within implantation can be explained by the combination of factors.

Autotransplantation of teeth also has a number of limitations. Most adult donor teeth are having formed root, which means that patients will need endodontic root canal treatment after transplantation. In addition, one of the most common complications of autotransplantation is root resorption and attachment loss (10). To prevent this complication, the use of a 3D printed model of a donor tooth is recommended to reduce the time it is out of the natural environment of the body and prevent drying of the periodontal ligament(11). Also, it should be noted that the anatomy of the transplanted tooth does not correspond to the removed one, which requires subsequent orthopedic treatment. These limitations confirm that autotransplantation can only be considered as an additional treatment option, with certain risks and complexities that need to be discussed with the patient in order to make a decision on the choice of treatment plan (3)

In presented clinical case, at the time of the patient’s treatment, significant bone atrophy and oroantral fistula were visualized, the initial conditions for the restoration by implantation were rather unfavorable. The plan of implantological treatment would include several surgical stages: elimination of the oroantral fistula, open sinus lifting using bone-plastic materials, in order to eliminate vertical bone loss in the distal part of a tooth 1.5, implantation itself, then installing the gingiva former, then permanent prosthetics. Summarizing: treatment would take at least one year and would involve certain risks at each stage of treatment(12). Autotransplantation of the third molar was presented as more gentle and more conservative when restoring an area with an oroantral communication.

Autotransplantation is considered the most predicTable option for replacing a lost tooth, as no plastic bone surgery needed. Studies have shown survival rates of 84%-94% of autotransplanted teeth (13).

Like any method of dental replacement, autotransplantation has its advantages and disadvantages. With a properly performed operation, the transplanted tooth functions as a normal one and can be even moved orthodontically (14), and also the operation carries minimal invasion for the body. But it should be taken into consideration that there is the statistical data on possible cervical resorption provided by Souza BDM, Dutra KL, Kuntze MM, Bortoluzzi EA (15). To exclude the possible risk of complications, it must be borne in mind that the manipulation must meet certain requirements: accurate measurements of the donor tooth and the receptive place must be performed, the removal of the donor tooth must be carried out carefully, minimal time outside of the socket for the donor tooth is a critical factor of autotransplantation success (13), similarly, when performing autotransplantation delayed, the receptive socket should be thoroughly cleaned of granulations.

The clinical case demonstrates the possibilities of autotransplantation.

This operation significantly reduces the duration of patient’s rehabilitation process, while ensuring minimal invasiveness: a gentle attitude to soft tissues, no need for bone plastic surgery, the possibility to use it as the base for orthopedic construction, also the tooth can be moved orthodontically.
